# Lethal and Pre-Lethal Effects of a Fungal Biopesticide Contribute to Substantial and Rapid Control of Malaria Vectors

**DOI:** 10.1371/journal.pone.0023591

**Published:** 2011-08-29

**Authors:** Simon Blanford, Wangpeng Shi, Riann Christian, James H. Marden, Lizette L. Koekemoer, Basil D. Brooke, Maureen Coetzee, Andrew F. Read, Matthew B. Thomas

**Affiliations:** 1 Center for Infectious Disease Dynamics, Mueller Laboratory, Department of Biology, Penn State University, University Park, Pennsylvania, United States of America; 2 Center for Infectious Disease Dynamics, Merkle Lab, Department of Entomology, Penn State University, University Park, Pennsylvania, United States of America; 3 Key Laboratory for Biological Control, China Agricultural University, Ministry of Agriculture, Beijing, China; 4 Vector Control Reference Unit, National Institute for Communicable Diseases of the National Health Laboratory Service, Johannesburg, South Africa; 5 Malaria Entomology Research Unit, School of Pathology, Faculty of Health Sciences, University of Witwatersrand, Johannesburg, South Africa; 6 Department of Biology, Mueller Laboratory, Penn State University, University Park, Pennsylvania, United States of America; 7 Fogarty International Center, National Institutes of Health, Bethesda, Maryland, United States of America; State University of Campinas, Brazil

## Abstract

Rapidly emerging insecticide resistance is creating an urgent need for new active ingredients to control the adult mosquitoes that vector malaria. Biopesticides based on the spores of entomopathogenic fungi have shown considerable promise by causing very substantial mortality within 7–14 days of exposure. This mortality will generate excellent malaria control if there is a high likelihood that mosquitoes contact fungi early in their adult lives. However, where contact rates are lower, as might result from poor pesticide coverage, some mosquitoes will contact fungi one or more feeding cycles after they acquire malaria, and so risk transmitting malaria before the fungus kills them. Critics have argued that ‘slow acting’ fungal biopesticides are, therefore, incapable of delivering malaria control in real-world contexts. Here, utilizing standard WHO laboratory protocols, we demonstrate effective action of a biopesticide much faster than previously reported. Specifically, we show that transient exposure to clay tiles sprayed with a candidate biopesticide comprising spores of a natural isolate of *Beauveria bassiana*, could reduce malaria transmission potential to zero within a feeding cycle. The effect resulted from a combination of high mortality and rapid fungal-induced reduction in feeding and flight capacity. Additionally, multiple insecticide-resistant lines from three key African malaria vector species were completely susceptible to fungus. Thus, fungal biopesticides can block transmission on a par with chemical insecticides, and can achieve this where chemical insecticides have little impact. These results support broadening the current vector control paradigm beyond fast-acting chemical toxins.

## Introduction

Current strategies for malaria control center on the use of chemical insecticides against the adult mosquito vectors [1]. Unfortunately, the sustainability and effectiveness of these frontline technologies is being undermined by the exceptionally rapid spread of insecticide resistance in *Anopheles* populations [2]. This growing resistance problem has led to calls for new control tools to help reduce the reliance on existing chemical insecticides [3]–[6].

A non-chemical approach that has received interest in recent years is the potential of fungal entomopathogens. The proposition is that these be formulated as biopesticides for use as indoor residual sprays or on treated materials and resting targets placed in and around the home. Since initial reports demonstrated the basic premise of this approach [7], [8], studies have explored the impact of fungal pathogens on the survival of a range of mosquitoes that vector disease [9]–[15], virulence against insecticide-resistant mosquitoes [16]–[18], possible methods of biopesticide delivery [19]–[20], and the impact of sub- and pre-lethal effects of infection on vectorial capacity [7], [21]. This largely laboratory-based empirical research has been supported by a number of modeling studies demonstrating the potential for use of fungal biopesticides in novel, sustainable integrated vector management strategies [22]–[25].

Unlike fast-acting chemical neurotoxins, fungal pathogens do not cause rapid mortality or immediate “knockdown” but rather act over a number of days as the fungal spores penetrate the insect cuticle and then proliferate within the hemocoel [26]. In certain agricultural applications, slow speed of kill has been identified as a constraint to biopesticide adoption [27]. This concern has also been raised with respect to malaria control [28], [29]. Indeed, the World Health Organization Pesticide Evaluation Scheme (WHOPES) approves for malaria control only insecticides that achieve greater than 80% mortality in twenty-four hours [30], [31], and others use this threshold to determine candidate compounds for inclusion in product development portfolios [5]. One important feature of malaria biology, however, is that the parasite typically takes 12–14 days to develop within the mosquito before it can be transmitted [32], [33] (and can take considerably longer depending on environmental conditions [34]). As such, even a slow acting biopesticide can halt transmission if mosquitoes contact fungal spores in one of their early feeding cycles; with WHO guidelines for IRS recommending treatment of at least 85% of houses [35], standard operational coverage should deliver this. This argument is founded on the fundamentals of malaria transmission [36] and is confirmed through detailed modeling studies [22]–[25].

Nonetheless, there exists a tension between conventional chemical paradigms, represented by the prevailing WHOPES criteria, which emphasize fast acting products for “mosquito control”, and what have been termed Late Life Acting products [24], which emphasize “malaria control” by reducing mosquito longevity.

Here we test the performance of a candidate biopesticide as if it were a conventional chemical insecticide. To date, no fungal studies have strictly followed WHOPES test protocols. We adopted the standard WHOPES assay methods [31] to test the residual action of a candidate fungal biopesticide applied to clay tiles. Clay is one of the standard substrates prescribed in the WHO guidelines and was selected here as the use of mud/clay in house construction is commonplace and its absorptive properties have proved challenging for conventional chemical insecticides [37]–[39]. We ask three questions. First, how do dose and exposure time affect efficacy on a natural substrate sprayed with a simple formulation of fungal spores? Second, what is the combined impact of lethal and pre-lethal effects of fungal infection on the capacity of mosquitoes to transmit malaria? Third, are insecticide resistant mosquitoes vulnerable to fungal attack? We find that through a combination of lethal and pre-lethal effects, the candidate biopesticide can produce extensive transmission blocking within a single feeding cycle. Additionally, we find that mosquitoes resistant to chemical insecticides are fully susceptible to fungus. Thus, from a disease control perspective, the biopesticide and existing chemical insecticides are similarly effective against susceptible mosquitoes, but the biological can sustain this performance against insecticide-resistant mosquitoes.

## Results

### Effect of substrate, application dose and exposure time

We followed the standard WHO ‘cone test’ methodology [31] to expose adult female *Anopheles stephensi* mosquitoes to clay tiles sprayed with an oil formulation of spores of the entomopathogenic fungus *Beauveria bassiana*. For the standard dose, spores were applied at an equivalent application rate of 5×10^11^ spores/m^2^ with mosquitoes exposed for 30 minutes one day after spraying. We first compared this dose with serial dilutions of 50%, 10%, 5% and 1%. We then varied exposure time, with periods reduced to 10, 5, 1 minute, and 30 seconds.

**Figure 1:**
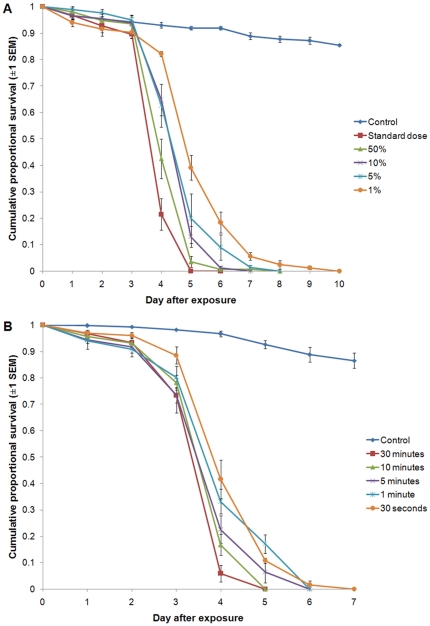
Cumulative proportional survival of *Anopheles stephensi*exposed to the standard dose (see main text) of *Beauveria bassiana* on clay for 30, 10, 5 or 1 minutes or 30 seconds (A); or for 30 minutes with doses reduced by 50, 10, 5 and 1% of the standard on clay tiles (B).

Median Lethal Time±95% confidence interval (MLT hereafter) of mosquitoes exposed to the standard dose was 4 (3.66–4.34) days, with 100% mortality occurring by day 5 (Fig. 1A). Reducing dose by up to 2 orders of magnitude had only moderate effects. At 50% dose there was no change in MLT and further dilutions increased MLT by one day only (see Supporting Information S1 for further details). 100% mortality ranged from 6–10 days across the declining doses (Fig. 1A).

Altering exposure period also had only moderate effects on mosquito survival. A 30 minute exposure resulted in 100% mortality in 5 days as did a 10 minute exposure. Reducing exposure times further to 5 minutes, 1 minute and 30 seconds resulted in 100% mortality in 6, 6 and 7 days respectively (Fig. 1B). All exposure times had the same MLT of 4 days (see Supporting Information S1). Overall, all doses and exposure periods were sufficient to reduce survivorship to at least 80% (the key WHOPES criterion) within 4–6 days.

### Survival of blood fed and sugar fed mosquitoes and propensity to feed

Impacting mosquito survival is one way of reducing vectorial capacity. However, fungal infection also reduces feeding propensity [7], [21]. If a mosquito won't feed, then it cannot transmit malaria. To investigate this pre-lethal effect under the current assay system we exposed mosquitoes to treated clay tiles (standard dose and exposure) and then monitored propensity to blood feed during the fungus incubation period. We examined mosquitoes maintained on glucose water only and mosquitoes that had just taken a blood feed.

**Figure 2:**
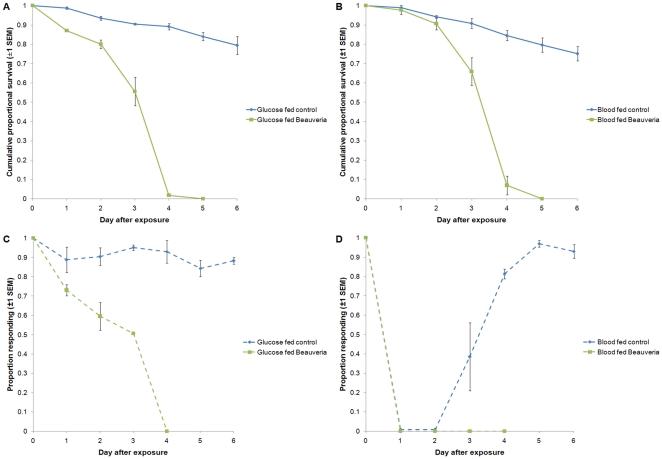
Survival and feeding propensity of *Anopheles stephensi* exposed to *Beauveria bassiana*. A) Survival of mosquitoes maintained on glucose only. B) Survival of blood fed mosquitoes. C) Daily mean proportion of glucose maintained mosquitoes responding to a feeding stimulus. D) Daily mean proportion of mosquitoes taking a blood meal (days 0 and 4) or responding to a feeding stimulus (days 1, 2 and 3).

In line with the baseline assays (Fig. 1), mosquitoes maintained on glucose water died rapidly with MLT of just 4 days (95% CI = 3.91–4.09) and 100% of all exposed insects dead by day 5. Over the same time period control mortality was 16±4.7% (Fig. 2A). When offered a feeding stimulus (see Materials and Methods), around 90% of control mosquitoes initiated feed behaviors on each day. In contrast, fungal exposed mosquitoes showed a declining response to the feeding stimulus over time, with 77, 60 and 50% of mosquitoes initiating feeding behaviors on days 1, 2 and 3, respectively and no mosquitoes responding on day 4 (repeated measures ANOVA gave a significant effect of both treatment, F_1,4_ = 94.98, *P* = 0.01, and time F_4,16_ = 75.86, *P*<0.001, on feeding propensity). Combining the proportion of mosquitoes alive with the proportion attempting to feed gives a measure of overall transmission blocking (biting risk) on any given day. For treated mosquitoes, this combination of pre-lethal and lethal effects revealed reductions in biting risk of 36, 52, 72 and 100% on days 1–4, respectively (Fig. 2B). This represents complete transmission blocking within a feeding cycle.

Adding the effects of blood feeding increased the effective impact of fungal infection further (Fig. 2C, D). While the survival pattern was very similar (control mortality was again 16±2.61% by day 5, and all fungal-exposed blood-fed mosquitoes dead by day 5 with an MLT of 4 days (95% C.I. = 3.92–4.08)) feeding response of all mosquitoes was strongly down regulated on days 1 and 2 following the blood feed, presumably because mosquitoes were digesting the blood meal and developing eggs. On day three, control mosquitoes showed increased responsiveness (38.4±17.6%) and on day 4, 81.4±2.6% of the control mosquitoes took a second blood meal (Fig. 2D). By contrast no fungal-exposed mosquitoes responded to the feeding stimulus on day 3 (survival at this point was 65.8±7.22%) and none of the surviving mosquitoes took a blood meal when it was offered on day 4 (Fig. 2D). Again this represents complete transmission blocking within a single feeding cycle; blood feeding essentially results in a “knockdown” in biting risk within 24 hrs from which fungal infected mosquitoes never recover.

### Delayed fungal exposures

One of the strengths of fast acting chemical insecticides is that malaria transmission can be blocked at any feeding cycle as long the mosquito is contacted before it becomes infectious. This contrasts with slow acting products that require contact early on in the parasite incubation period (see earlier arguments). However, while fungal biopesticides have traditionally been viewed as slow acting, the results from the assays above demonstrate that, in fact, transmission blocking can be very rapid. In principle, this should enable effective malaria control even if mosquitoes escape fungal infection until late in life. To investigate this we exposed a group of mosquitoes to fungal treated clay tiles on day 0 as (above) but also included groups that were not exposed to treated tiles until the second (day 4), third (day 8), or fourth (day 12) feeding cycles. At all other times fungal treatment groups and the controls were exposed to untreated tiles. All groups were offered a blood meal (as above) on day 0 and every four days subsequently up to and including day 16. In between blood meals the insects' propensity to feed was assessed using the same feeding stimulus described above.

**Figure 3:**
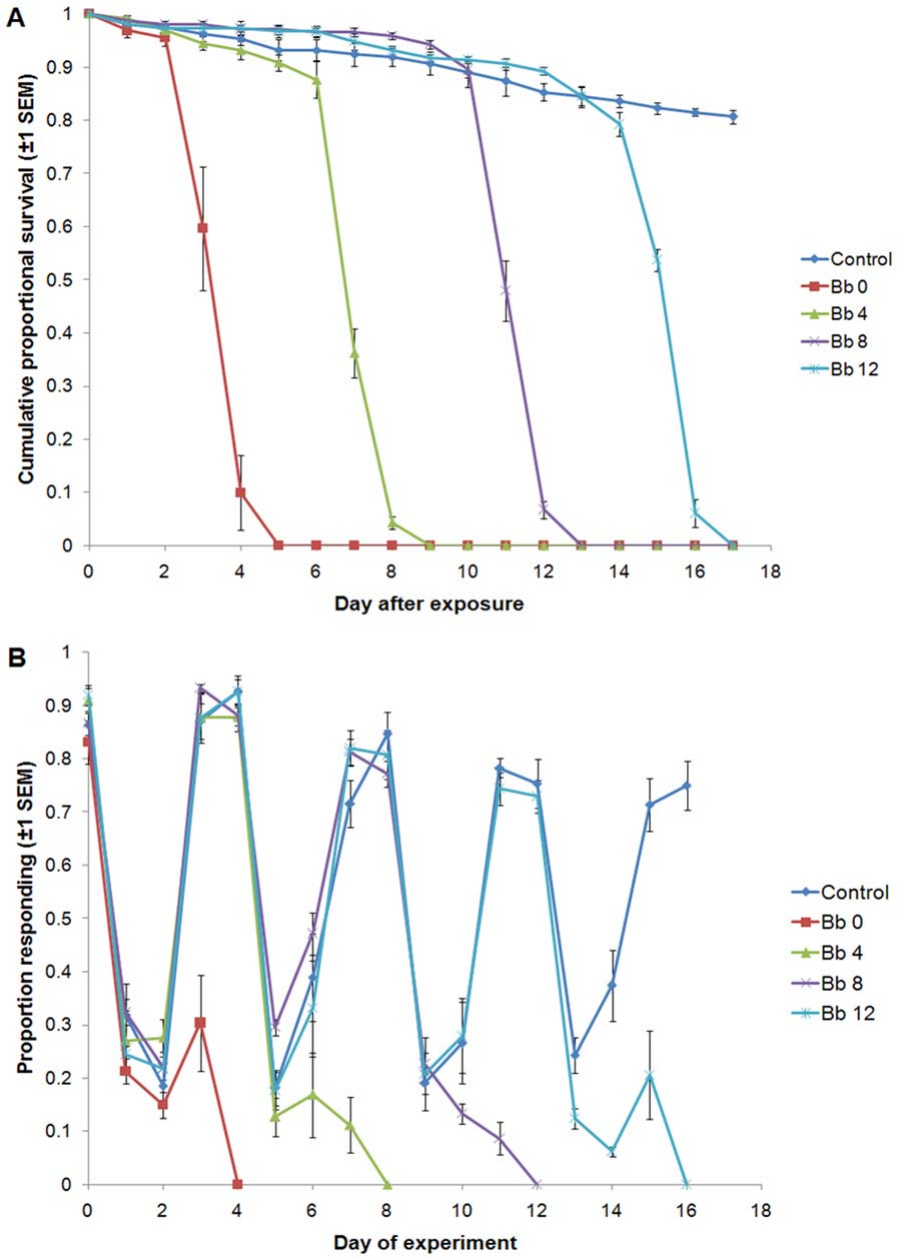
A) Survival of *Anopheles stephensi* exposed to clay tiles treated with *Beauveria bassiana* on either day 0 (Bb 0), or not until day (Bb 4), day 8 (Bb 8) or day 12 (Bb 12). Control mosquitoes were exposed to untreated clay tiles on each of these days. B) Proportion of mosquitoes taking a blood meal on days 0, 4, 8, 12 and 16 and proportion responding to a feeding stimulus in between the blood meal days.

The control group had very little mortality with 80.6 (±1.24)% surviving to the end of the experiment. Median lethal times (±95% C.I.) for the fungal exposures were 4 (3.92–4.08), 7 (6.88–7.12), 11 (10.85–11.15) and 16 (15.92–16.08) days for the groups exposed to the fungi on days 0, 4, 8, and 12 respectively (Fig. 3A). When survival of fungal groups was assessed from the day of exposure, median lethal times were 4 (3.92–4.08), 3 (2.88–3.12), 3 (2.85–3.15) and 4 (3.94–4.06) days for the day 0, 4, 8, and 12 day exposure groups respectively. All fungal exposed mosquitoes in each treatment group were dead within 5 days of exposure time.

Across all blood feeding episodes a minimum of 75% of the control mosquitoes took a blood meal (Fig. 3B). The pattern of response to the feeding stimulus between blood meals was not as clear cut as in the previous assay as we did not clear non-fed and partially fed mosquitoes from the cages. Hence even on the first day after each blood feed some mosquitoes were probing at the feeding stimulus. Nevertheless, the general pattern was similar with control insects showing a sharp decline in feeding propensity in the two days following a blood meal (generally less than 40% responded - Fig. 3B) followed by a return to the feeding stimulus by day 3 or 4. In contrast, all the fungal-exposed treatments showed substantial reductions in feeding propensity, with no mosquitoes taking a blood meal at 4 days after fungal exposure (see Fig. 3B).

Interestingly, despite the rapid impact on survival and feeding, fungal-exposed insects still realized some fecundity. In the gonotrophic cycle following exposure, the day 0, 4, 8, and 12 day exposure groups laid 59, 53, 53 and 75% of the number eggs produced by the relative controls, respectively.

### Metabolic rate and tethered flight performance

**Figure 4:**
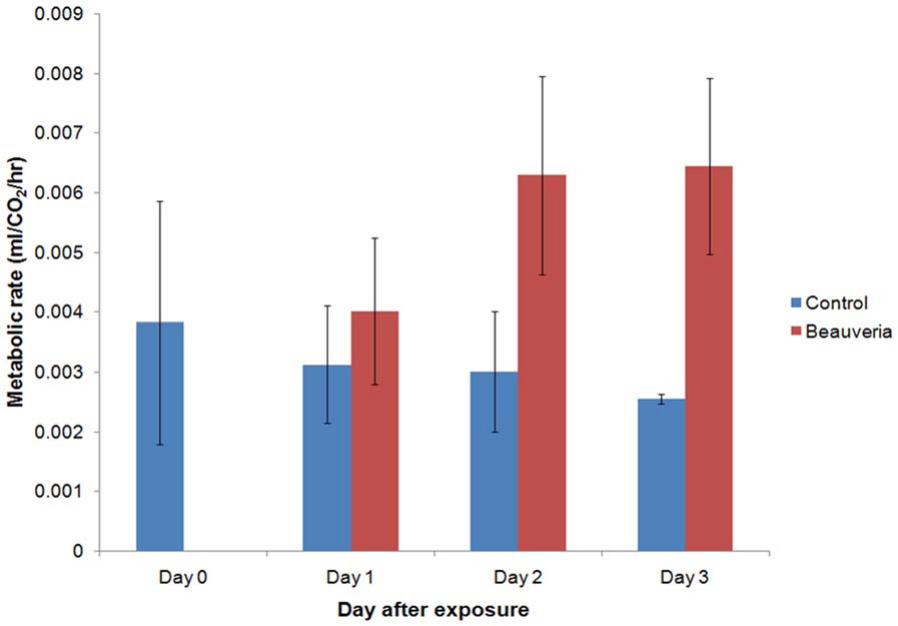
Resting metabolic rate of control and fungal exposed *Anopheles stephensi* as measured by CO_2_ output in flow through respirometer. A sample of mosquitoes used in the experiment was assayed for metabolic rate on day 0 to provide a pre-treatment comparison. The measurement variable is CO_2_ emmission (ml/hr) as body size had no detectable effect on CO_2_ output.

The pre-lethal effects of fungal infection on feeding propensity are clearly substantial and important. However, given the nature of the fungal infection process (i.e. physical proliferation within the insect together with production of various secondary metabolites known to impact aspects of insect physiology [40]–[42]) it is reasonable to expect that an infected mosquito might suffer additional reductions in performance prior to death. One way of looking at the gross impact of infection is to measure respiration via CO_2_ output as a proxy for resting metabolic rate (RMR). Here, we measured RMR of uninfected and infected mosquitoes using a flow-through respirometer (see Materials and Methods for full description). Prior to exposure, metabolic rates of a sub-sample of mosquitoes assigned to control and treatment groups were measured. This baseline RMR did not differ significantly to metabolic rates of control insects measured over the following three days (F_3,24_ = 0.21 *P* = 0.89: Fig 4a). Fungal-infected mosquitoes, on the other hand, showed a significant elevation in metabolic rate (F_1,33_ = 6.31, *P* = 0.017), 2–2.5 times greater than controls (Fig. 4a). Mortality in the treated group left insufficient insects to do a full run on day 4. No significant effects of insect size, time of day, respirometer chamber, day after exposure, or ‘day×treatment’ interaction were observed (see Supporting Information S1).

**Figure 5:**
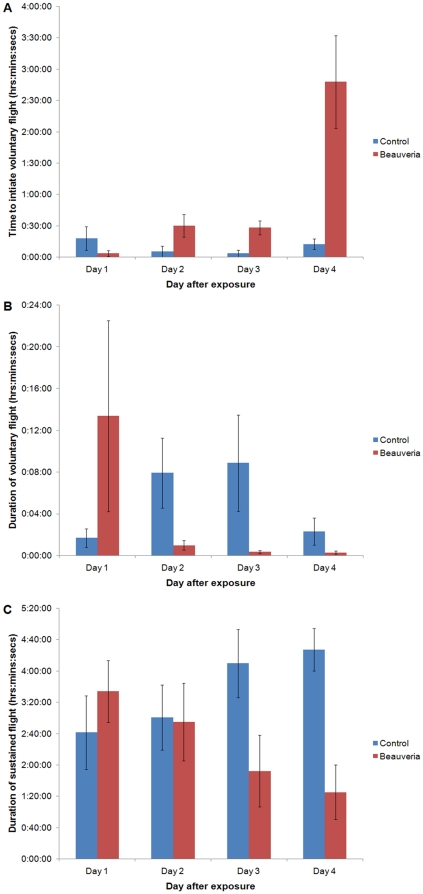
Tethered flight performance of control and fungal exposed *Anopheles stephensi*. A) Time to start of voluntary flight and B) the length of that initial flight. C) The duration flight was maintained in mosquitoes repeatedly stimulated.

The full implications of the elevated RMR are unclear, but the increased energy expenditure might be expected to impact on energetically costly behaviors such as flight. To investigate this we suspended infected and uninfected female *An. stephensi* mosquitoes on fine insect pins and measured three aspects of flight performance (Fig. 5). We found that the time to initiate voluntary flight increased in fungal-exposed insects, becoming significantly longer than controls from day 2 after exposure (Mann-Whitney U = 11, d.f. 18, z = −2.66, *P* = 0.008) (Fig. 5A). By day 4, only half of the treated insects initiated flight during the whole 5 hour monitoring period, whereas all control insects flew. Additionally, the length of the first voluntary flight was significantly shorter in fungal exposed insects from day 2 onwards (*P* = 0.003 - see Supporting Information S1 for full statistics) (Fig. 5B). Finally, the ability to sustain flight following repeated physical stimulation (i.e. flight to exhaustion, providing a measure of the energetic budget available to the insect) was also significantly shorter in treated insects. By day 3 fungal exposed insects could fly for only half as long as control insects (*P* = 0.009) and by day 4 this was reduced further to just a third (*P* = 0.015; Fig. 5C).

### Efficacy against insecticide resistant mosquitoes

**Figure 6:**
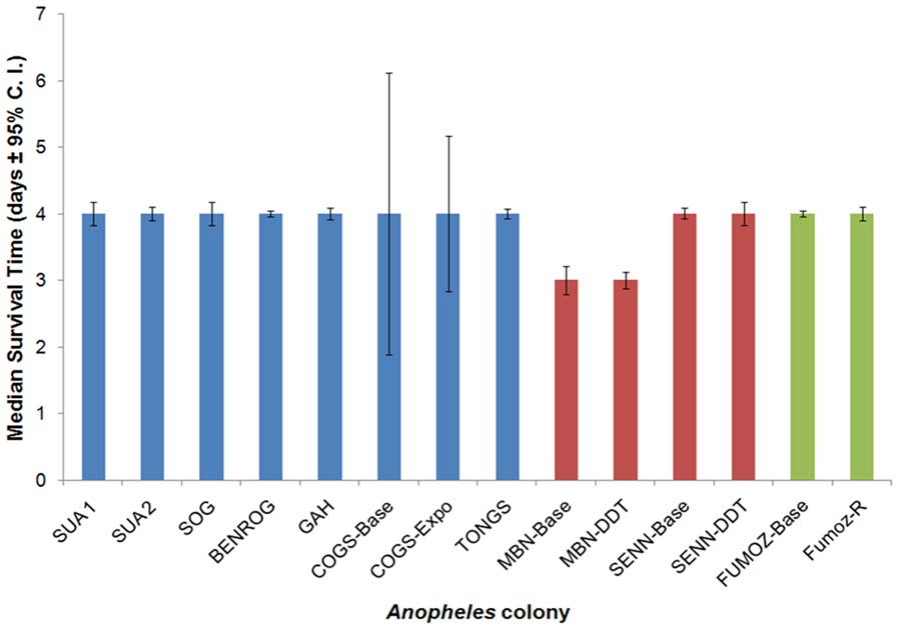
Survival of thirteen colonies of *Anopheles* mosquitoes following exposure to *Beauveria bassiana* on clay tiles. Resistance to chemical insecticides for each colony was determined and ranged between fully susceptible and resistant to all four classes of chemical insecticides currently used for vector control (see main text and Supporting Information S1). Two assays were conducted with the fully susceptible *Anopheles gambiae* s.s. SUA colony acting as a positive control in each assay. Blue bars are *Anopheles gambiae* s.s., red bars *Anopheles arabiensis* and green bars *Anopheles funestus* colonies.

To assess the impact of the fungal biopesticide on mosquitoes resistant to chemical insecticides we ran two bioassays against thirteen colonies of *Anopheles* mosquitoes comprising three different species (*An. gambiae* s.s., *An. arabiensis* and *An. funestus*). The colonies were assessed for their resistance to four compounds (an organophosphate, organochloride, pyrethroid and carbamate) at discriminatory doses prescribed by WHO [31] using a standard cylinder assay. The colonies were found to range from fully susceptible, resistant to one of the compounds, to two, three or in one case all four chemical classes (see Supporting Information S1 for colony description and details of the insecticides and assay performed). Exposure to the fungal biopesticide on clay tiles using the standard dose and thirty minute exposure period as described above showed MLTs of three or four days (see Fig. 6) and 100% mortality by day 6 irrespective of mosquito species or the level of chemical resistance. For example, the *An. gambiae* colony “TONGS”, which was fully resistant to all chemical classes, had an MLT of 4 (3.93–4.07) days and all individuals were dead by day 5 (±0.0) which was not dissimilar to the fully susceptible *An. gambiae* colony “SUA” which had an MLT of 4 (3.82–4.18) days and were all dead by day 6 (±0.25) (see Supporting Information S1 for further details).

## Discussion

The rapid mortality of mosquitoes following short-term residual contact with fungal spores on a realistic substrate is much faster than reported in many earlier studies [7], [8], [10], [13], [16], [18]. We are not sure why this should be; the fungal isolate has been tested before, and at the doses we used here. What is different here is that the exposures were made on clay substrates in a cone test and that the spray apparatus used appears to result in more efficient delivery of spores to the substrate ([43] The use of this assay system, which is that recommended by WHO, suggests that previous reports of efficacy were underestimates).

As indicated previously, the average feeding cycle of *Anopheles* mosquitoes in the field is 2–4 days and the incubation period of the malaria parasite inside the mosquito at least 12–14 days. In order for a mosquito to transmit malaria, therefore, it needs to pick up the parasite from an infected human host and survive 3 or 4 subsequent feeding cycles before being able to transmit the parasite to another human host. Contacting a lethal insecticide like permethrin or DDT at any one of these cycles would stop transmission – which is why properly implemented ITN and IRS programs work so well (at least in the absence of resistance). While still not an instant knockdown, our results indicate that if mosquitoes contacted a virulent fungus at any feeding cycle within the parasite incubation period the mosquitoes would not survive long enough to transmit malaria either.

Insect death, however, is only part of the story. Our results also show that once infected with fungus, mosquitoes are less inclined to feed. The effect appears stronger as the fungal infection progresses but can contribute to significant reductions in host feeding as early as day two, essentially accelerating the transmission blocking effects of the fungus. Pre-lethal reductions in feeding propensity have been shown before [7], [21] and appear a common effect of fungal infection in insects [44]–[47]. What is striking here is that when the effects of blood feeding are added in, risk of malaria transmission is essentially reduced to zero within a day of fungal exposure and never recovers.

In addition, fungal infection increases mosquito metabolic rate and reduces flight propensity and flight stamina. Again, fungal induced reductions in flight performance and elevated metabolic rate have been shown previously in other insects [47]–[50] and poor flight performance has been strongly associated with reductions in the mobile energy reserves of the host [48]. Energetic demands in the field associated with host finding, searching for oviposition sites and predator avoidance (previously shown to be compromised in fungal-infected insects [51]) are likely to be considerably higher than in our laboratory setting. We have been dealing with young, healthy insects maintained under ideal conditions. Long range flights to search for nectar sources, blood meals and oviposition sites under variable environmental conditions [52], [53], coupled with the agility required to evade death while blood feeding and repeated contact with treated surfaces, makes life hard for a mosquito. Add in the burden of malaria infection [54]–[58] and age-related senescence [59]–[63] then there is every reason to think that the behavioral, physiological and survival effects we have found so far are underestimates of the potential of fungi to reduce malaria transmission. Indeed, it is an interesting possibility that in the natural context, simply making mosquitoes sick could be sufficient to disrupt the malaria transmission cycle.

Our comprehensive evaluation of multiple mosquito strains and species covering diverse mechanisms and expressions of insecticide resistance demonstrates that insecticide resistance confers no cross-resistance to fungal pathogens in the key African malaria vectors. This result extends previous studies [16], [17] and contrasts the situation with chemical insecticides where there are major problems of cross-resistance, undermining the potential of many resistance management strategies [2], [4]. Furthermore, co-exposure to fungus and insecticide has been shown to increase the susceptibility of otherwise resistant mosquitoes to existing chemical insecticides [16], [17].

Several previous studies have argued for the evolutionary benefits of Late Life Acting products; slow speed of kill enables mosquitoes to achieve part of their lifetime reproductive output, reducing selection pressure for resistance [24]–[26]. In a very recent development, Fang et al. [64] demonstrated the potential to genetically modify an insect pathogenic fungus to kill malaria parasites within mosquitoes without dramatically impacting mosquito survival, further extending the ‘evolution proof’ potential. In our study, the fungus clearly imposes a fitness cost, although the fact that there is some realized fecundity following infection suggests there would still be reduced selection pressure for resistance compared with the instantaneous action of conventional chemicals [24].

While the resistance management benefits of Late Life Acting products are clear in theory, the approach has been criticized as impractical [28], [37] and sits at odds with current WHOPES criteria. Acknowledging these issues, the current study attempts to align the fungal biopesticide approach with the established chemical insecticide paradigm. Our results demonstrate that using the standard WHOPES protocols, short-term residual contact with fungal spores on a realistic substrate can cause extensive mortality of mosquitoes. Important pre-lethal effects including reduced feeding propensity and flight stamina reduce vectorial capacity further; if a mosquito does not want to feed, is less able to sustain flight to search for a host and ultimately dies before the malaria parasite can complete its development, there will be no transmission. Thus the combined pre-lethal and lethal effects of fungi, together with resistance breaking properties, make possible malaria control without fast acting neurotoxins (reasserting the fact that other once successful IRS treatments, such as cyclodiene insecticides in the 1950s, were slower acting [65]). While it would also be possible to enhance speed of kill further by genetically modifying fungi [64], [66], the data we report here suggests that natural variation alone may be sufficient.

The fungal isolate used in the current study exists as a commercial pest control product and has full EU and US registration for certain agricultural applications [67], [68]. In principle, repurposing this isolate for malaria control could deliver a novel product for operational use in a relatively short timeframe, and at a fraction of the R&D costs of a new chemical entity. Given the emerging insecticide resistance crisis and acknowledged difficulties in getting new chemical insecticides to the market for many years [2], further evaluation of the fungal biopesticide approach under diverse field conditions would seem justified.

## Materials and Methods

### Ethics statement

This study was carried out in strict accordance with the recommendations in the Guide for the Care and Use of Laboratory Animals of the National Institutes of Health. The protocol was approved by the Animal Care and Use Committee of the Pennsylvania State University (Permit Number: 27452). All mice were anesthetized prior to mosquito feeds using a Xylazine∶Ketamine (0.15∶1) mix at 0.1 ml/10 grams body weight i.p. and all efforts were made to minimize suffering.

### Fungal application, mosquito exposure and survival monitoring

*Beauveria bassiana* spores were formulated in a mix of mineral oils and the concentrations adjusted to give the desired spores/ml of formulation. Formulated spores were applied using a pump sprayer clamped horizontally over the test tile and 10 cm above it. Each tile received five pumps from the sprayer which delivered 0.7 ml of formulation with each pump. Following application tiles were left to dry at room temperature for 24 hours. A standard WHO cone assay was used for exposing the mosquitoes to the treated tiles. The plastic cone was secured over each tile and between 30 and 60 (depending on assay) unfed female *An. stephensi* were introduced. The mosquitoes were then left for 30 seconds, 1 minute, five minutes, 10 minutes or 30 minutes depending on the assay. Each treatment was replicated four times giving a minimum of 120 mosquitoes per assay. Following exposure mosquitoes were removed to holding cages were they were either blood fed (see below) or maintained on 10% glucose water for the duration. Subsequently each day either until all treated insects had died or for 14 days, which ever was earlier, the number of dead mosquitoes were counted and removed from the cages.

### Feeding propensity

Two methods were used to assess feeding propensity. Mosquitoes were offered either an anaesthetized mouse or a glass bottle filled with hot water (temperature range 35–42°C) and then covered with an investigator's recently worn sock. The sock covered bottle was placed next to the side of the cage and in contact with the mesh. On the day of blood feeds, mosquitoes were allowed to feed from mice for twenty minutes and then those not taking a blood meal were counted. On intervening days the “sock-bottle” method was used as a feeding stimulus and the number not actively probing at the bottle were counted. In both cases this number was related to the total number of mosquitoes in that cage at the time of the feeding assessment to give the proportion feeding on that day.

### Fecundity assessment

Assays where anaesthetized mice were used for feeding also provided the opportunity to assess fecundity. Two days following the first blood meal egg bowls (35 mm Petri dishes lined with filter paper and soaked with distilled water) were introduced. The following morning these bowls were removed and replaced with fresh bowls and this was repeated until the end of the experiment. Eggs were counted and related to the number of mosquitoes alive to give an eggs/female fecundity estimate.

### Estimating Resting Metabolic Rate

Metabolic rates were measured by using groups of three mosquitoes at rest within flow-through respirometry chambers. Dry CO_2_ free air was passed through the 20 ml chambers at 0.25 litres/min and then dried and passed through a Li-Cor 6252 carbon dioxide analyser. Within each run, seven experimental chambers containing mosquitoes were sampled in sequential fashion by using a computer controlled valve system. Three chambers containing control mosquitoes and four for fungal exposed mosquitoes were used in the first run and the order was reversed for the second run giving 7 replicate estimates per day of the experiment. An eighth chamber was left empty and sampled between each of the occupied chambers to establish a baseline. A pre-treatment run was performed on day 0 using the mosquitoes to be allocated to treatment groups and then two runs were made every day for the duration of the experiment. All chambers were housed in a reach-in incubator set to 25 (±0.2)°C. Analog signals from the flow meter and carbon dioxide analyzer were converted to digital and recorded on a computer (Sable Systems, Salt Lake City).

### Flight performance

To assess flight performance female mosquitoes were briefly immobilized with CO_2_ and then placed on a dish under a dry ice curtain. A small amount of dental wax was melted on the blade of a surgical scalpel and the head of an insect pin touched to the wax. Immediately following a mosquito gently held in forceps was manipulated so that the dorsal thorax came into contact with the head of the pin, held briefly to allow the wax to set and then the sharp end of the pin was stuck vertically in a block of polystyrene. Each mosquito was carefully examined to ensure that no wax had spread over the insect, that no damage to the insect had been caused and that the insects' orientation was appropriate (i.e. not cantered to one side, forward or back). In any case where the mounting of the insect on the pin did not satisfy these requirements the mosquito was discarded and a new one set up. Two measures were made: 1) the duration of voluntary flight. In this case the time until first flight was initiated was recorded as well as the duration of this flight. The second measure looked at the duration of sustained flight. In this case and following the end of the voluntary flight the mosquito was stimulated to fly again by gently stroking its legs with a fine paint brush. Each time the mosquito stopped flying it was stimulated again until after five consecutive stimulations the insect could not fly again. Flight assays were censored after five hours.

### Assays against chemical resistant mosquitoes

Assays were carried out at the VCRU/NICD/NHLS facility in South Africa where a range of resistant and susceptible *Anopheles* colonies are maintained. Two assays were performed. In each a fully susceptible *Anopheles gambiae* s.s. colony (SUA) was included. Again the WHO cone test was employed and all methodologies, substrate, standard dose, application method and exposure time were as described above. In each assay and following exposure to either untreated or *Beauveria* treated clay tiles mosquitoes were removed to 0.375 ml cardboard cups covered with mesh and supplied with 10% sucrose. Mortality was monitored daily for 14 days. Following the fungal assays mosquitoes from each colony used were exposed to each of four chemical insecticides (DDT, Bendiocarb, Malathion and Deltamethrin) representing the four major classes of compound available for vector control (Organochlorides, Carbamates, Organophosphates and Pyrethroids). A standard WHO cylinder assay was used with four replicates of 25 mosquitoes per replicate for each colony used on the fungal assays. Mortality was assessed after 24 hours and related to the WHO criteria for assessing resistance/susceptibility to chemical insecticides. Further details can be found in the supplementary materials.

## Supporting Information

Supporting Information S1 **Further details of the material and methods, analyses and results for the experiments described in the main text.**
(DOC)

## References

[1] Global Malaria Action Plan website.. http://www.rollbackmalaria.org/gmap/gmap.pdf.

[2] Ranson H, N'Guessan R, Lines J, Moiroux N, Nkuni Z (2011). Pyrethroid resistance in African anopheline mosquitoes: what are the implications for malaria control?. Trends Parasitol.

[3] Zaim M, Guillet P (2002). Alternative insecticides: an urgent need.. Trends Parasitol.

[4] Nauen R (2007). Insecticide resistance in disease vectors of public health importance.. Pest Manag Sci.

[5] Hemingway J, Beaty BJ, Rowland M, Scott TW, Sharp BL (2006). The innovative vector control consortium: improved control of mosquito-borne diseases.. Trends Parasitol.

[6] Kelly-Hope L, Ranson H, Hemingway J (2008). Lessons from the past: managing insecticide resistance in malaria control and eradication programmes.. Lancet Infect Dis.

[7] Blanford S, Chan BHK, Jenkins N, Sim D, Turner RJ (2005). Fungal pathogen reduces potential for malaria transmission.. Science.

[8] Scholte E-J, Ng'Habi K, Kihonda J, Takken W, Paaijmans KP (2005). An entomopathogenic fungus for control of adult African malaria mosquitoes.. Science.

[9] Scholte EJ, Takken W, Knols BGJ (2007). Infection of adult *Aedes aegypti* and *Ae. albopictus* mosquitoes with the entomopathogenic fungis *Metarhizium anisopliae*.. Acta Tropica.

[10] Achonduh OA, Tondje PR (2008). First report of pathogenicity of *Beauveria bassiana* RBL1034 to the malaria vector, *Anopheles gambiae* s.l. (Diptera: Culicidae) in Cameroon.. African J Biotechnol.

[11] Mohanty SS, Raghavendra K, Rai U, Dash AP (2008). Efficacy of female *Culex quinquefasciatus* with entomopathogenic fungus *Fusarium pallidoroseum*.. Parasitol Res.

[12] De Paula AR, Brito ES, Pereira CR, Carrera MP, Samuels RI (2008). Susceptibility of adult *Aedes aegypti* (Diptera: Culicidae) to infection with *Metarhizium anisopliae* and *Beauveria bassiana*: prospects for Dengue control.. Biocontrol Science and Techn.

[13] Mnyone LL, Russell TL, Lyimo IN, Lwetoijera DW, Kirby MJ (2009). First report of *Metarhizium anisopliae* IP 46 pathogenicity tin adult *Anopheles gambiae* s.s. and *An. arabiensis* (Diptera: Culicidae).. Parasite Vector.

[14] Mnyone LL, Kirby MJ, Lwetoijera DW, Mpinga MW, Knols BGJ (2009). Infection of the malaria mosquito, *Anopheles gambiae*, with two species of entomopathogenic fungi: effects of concentration, formulation, exposure time and persistence.. Malaria J.

[15] Farenhorst M, Knols BGJ (2010). A novel method for standardized application of fungal spore coatings for mosquito exposure bioassays.. Malaria J.

[16] Farenhorst M, Mouatcho JC, Kikankie CK, Brooke BD, Hunt RC (2009). Fungal infection counters insecticide resistance in African malaria mosquitoes.. Proc Natl Acad Sci USA.

[17] Farenhorst M, Knols BGJ, Thomas MB, Howard AFV, Takken W (2010). Synergy of fungal entomopathogens and permethrin against West African insecticide-resistant *Anopheles gambiae* mosquitoes.. PLoS One.

[18] Kikankie CK, Brooke BD, Knols BGJ, Koekemoer LL, Farenhorst M (2010). The infectivity of the entomopathogenic fungus *Beauveria bassiana* to insecticide-resistant and susceptible *Anopheles arabiensis* at two different temperatures.. Malaria J.

[19] Lwetoijera DW, Sumaye RD, Madumla EP, Kavishe DR, Mnyone LL (2010). An extra-domiciliary method of delivering entomopathogenic fungus, *Metarhizium anisopliae* IP 46 for controlling adult populations of the malaria vector, *Anopheles arabiensis*.. Parasite Vector.

[20] Farenhorst M, Farina D, Scholte E-J, Takken W, Hunt RH (2008). African water storage pots for the delivery of the entomopathogenic fungus *Metarhizium anisopliae* to the malaria vectors *Anopheles gambiae* s.s. and *Anopheles funestus*.. Am J Trop Med Hyg.

[21] Scholte EJ, Knols BGJ, Takken W (2006). Infection of the malaria mosquito *Anopheles gambiae* with the entomopathogenic fungus *Metarhizium anisopliae* reduces blood feeding and fecundity.. J Invert Pathol.

[22] Hancock PA (2009). Combining fungal biopesticides and insecticide-treated bednets to enhance malaria control.. PLoS Comput Biol.

[23] Hancock PA, Thomas MB, Godfray HCJ (2009). An age-structured model to evaluate the potential of novel malaria-control interventions: a case study of fungal biopesticide sprays.. P Roy Soc Lond B Bio.

[24] Read AF, Lynch PA, Thomas MB (2009). How to make evolution-proof insecticides for malaria control.. PLoS Biol.

[25] Koella JC, Lynch PA, Thomas MB, Read AF (2009). Towards evolution-proof malaria control with insecticides.. Evol Appl.

[26] Thomas MB, Read AF (2007). Can fungal biopesticides control malaria?. Nat Rev Microbiol.

[27] Lacey LA, Frutos R, Kaya HK, Vail P (2001). Insect pathogens as biological control agents: do they have a future?. Biol Control.

[28] Dolgin E (2009). Evolution, Resisted.. The Scientist.

[29] Hutchinson OC, Cunningham AA (2005). Benefits and risks in malaria control.. Science.

[30] World Health Organization website.. http://www.who.int/whopes/gcdpp/publications/en/index2.html.

[31] World Health Organization website.. http://www.who.int/whopes/gcdpp/publications/en/index1.html.

[32] Charlwood JD, Smith T, Billingsley PF, Takken W, Lyimo EOK (1997). Survival and infection probabilities of anthropophagic anophelines from an area of high prevalence of *Plasmodium falciparum* in humans.. Bull Ent Res.

[33] Killeen GF, McKenzie FE, Foy BD, Schieffelin C, Billingsley PF (2000). A simplified model for predicting malaria entomologic inoculation rates based on entomologic and parasitologic parameters relevant to control.. Am J Trop Med Hygiene.

[34] Paaijmans KP, Read AF, Thomas MB (2009). Understanding the link between malaria risk and climate.. P Natl Acad Sci USA.

[35] World Health Organization website.. http://whqlibdoc.who.int/publications/2010/9789241564106_eng.pdf.

[36] Boyd MF (1949). In Malariology: A comprehensive survey of all aspects of this group of diseases from a global standpoint ed..

[37] World Health Organisation website.. http://whqlibdoc.who.int/hq/2001/WHO_CDS_WHOPES_2001.3.pdf.

[38] Singh K, Rahman SJ, Joshi GC (1989). Village scale trials of deltamethrin against mosquitoes.. J Commun Dis.

[39] Vantandoost MR, Abai M, Shaegi M, Abtahi M, Rafie F (2009). Designing of a laboratory model for evaluation of the residual effects of deltamethrin (K-othrine WP 5%) on different surfaces against malaria vector, *Anopheles stephensi* (Diptera: Culicidae).. J Vector Dis.

[40] Kershaw MJ, Moorehouse ER, Bateman R, Reynolds SE, Charnley AK (1999). The role of destruxins in the pathogenicity of *Metarhizium anisopliae* for three species of insect.. J Invert Pathol.

[41] Hung SY, Boucias DG (1992). Influence of *Beauveria bassiana* on the cellular defense response of the beet armyworm, *Spodoptera exigua*.. J Invert Pathol.

[42] Hajek AE, St Leger RJ (1994). Interactions between fungal pathogens and insect hosts.. Annu Rev Entomol.

[43] Bell AS, Blanford S, Jenkins N, Thomas MB, Read AF (2009). Real-time quantitative PCR analysis of candidate fungal biopesticides against malaria: Technique validation and first applications.. J Invert Pathol.

[44] Tefera T, Pringle KL (2003). Food consumption by *Chilo partellus* (Lepidoptera: Pyralidae) larvae infected with *Beauveria bassiana* and *Metarhizium anisopliae* and effects of natural versus artificial diets on mortality and mycosis.. J Invert Pathol.

[45] Thomas MB, Blanford S, Gbongbui C, Lomer CJ (1998). Experimental studies to evaluate spray applications of a mycoinsecticide against the rice grasshopper, *Hieroglyphus danganesis*, in northern Benin.. Entomol Exp Applic.

[46] Arthurs SA, Thomas MB (2001). Effects of a mycoinsecticide on feeding and fecundity of the brown locust, *Locustana pardalina*.. Biocontr Sci Technol.

[47] Seyoum E, Moore D, Charnley AK (1994). Reduction in flight activity and food consumption by the desert locust, *Schistocerca gregaria*, after infection with *Metarhizium flavoviride*.. J Appl Entomol.

[48] Seyoum E, Bateman RP, Charnley AK (2002). The effect of *Metarhizium anisopliae* var *acridum* on haemolymph energy reserves and flight capability in the desert locust, *Shistocerca greagaria*.. J Appl Entomol.

[49] Wiygul G, Sikorowski PP (1981). Oxygen uptake in larval bollworms (*Heliothis zea*) infected with the fungus *Nomurea rileyi*.. Comp Biochem Physiol.

[50] Sewify GH, Hashem MY (2001). Effects of the entomopathogenic fungus *Metarhizium anisopliae* (Metsch.) Sorokin on cellular defence response and oxygen uptake of the wax moth *Galleria mellonella* L. (Lep., Pyralidae).. J Appl Entomol.

[51] Arthurs SA, Thomas MB (2001). Investigation into behavioral changes in *Schistocerca gregaria* following infection with a mycoinsecticide: implications for susceptibility to predation.. Ecol Entomol.

[52] Gillies MT (1961). Studies on the dispersion and survival of *Anopheles gambiae* Giles In East Africa, by means of marking and release experiments.. Bull Entomol Res.

[53] Costantini C, Li SG, DellaTorre A, Sagnon N, Coluzzi M (1996). Density, survival and dispersal of *Anopheles gambiae* complex mosquitoes in a West African Sudan savanna village.. Med Vet Entomol.

[54] Ferguson HM, Read AF (2002). Why is the impact of malaria parasites on mosquito survival still unresolved?. Trends Parasitol.

[55] Rivero A, Ferguson HM (2003). The energetic budget of *Anopheles stephensi* infected with *Plasmodium chabaudi*: is energy depletion a mechanism for virulence?. P Roy Soc Lond B Bio.

[56] Lambrechts L, Chavatte JM, Snounou G, Koella JC (2006). Environmental influences on the genetic basis of mosquito resistance to malaria parasites.. P Roy Soc Lond B Bio.

[57] Ahmed AM, Hurd H (2006). Immune stimulation and malaria infection impose reproductive costs in *Anopheles gambiae* via follicular apoptosis.. Microbes Infect.

[58] Hurd H (2007). Nature or nurture in mosquito resistance to malaria?. Trends Parasitol.

[59] Rowland M, Hemingway J (1987). Change in malathion resistance with age in *Anopheles stephensi* from Pakistan.. Pest Biochem Physiol.

[60] Lines JD, Nassor NS (1991). DDT resistance in *Anopheles gambiae* declines with mosquito age.. Med Vet Entomol.

[61] Hunt RH, Brooke BD, Pillay C, Koekemoer LL, Coetzee M (2005). Laboratory selection for and characteristics of pyrethroid resistance in the malaria vector *Anopheles funestus*.. Med Vet Entomol.

[62] Hodjati MH, Curtis CF (1999). Evaluation of the effects of mosquito age and prior exposure to insecticide on pyrethroid tolerance in *Anopheles* mosquitoes (Diptera: Culicidae).. Bull Entomol Res.

[63] Matambo TS, Abdalla H, Brooke BD, Koekemoer LL, Mnzava A (2007). Insecticide resistance in the malarial mosquito *Anopheles arabiensis* and association with the kdr mutations.. Med Vet Entomol.

[64] Fang W, Vega-Rodriguez J, Ghosh AK, Jacobs-Lorena M, Kang A (2011). Development of transgenic fungi that kill human malaria parasites in mosquitoes.. Science.

[65] Oliver SV, Kaiser ML, Wood OR, Coetzee M, Rowland M (2010). Evaluation of the pyrrole insecticide chlorfenapyr against pyrethroid resistant and susceptible *Anopheles funestus* (Diptera: Culicidae).. Trop Med Int Health.

[66] Weng C, St Leger RJ (2007). A scorpion neurotoxin increases the potency of a fungal insecticide.. Nat Biotechnol.

[67] Environmental Protection Agency website.. http://www.epa.gov/oppbppd1/biopesticides/ingredients/tech_docs/tech_128924.htm.

[68] Pesticide Action Network (PAN) Pesticide database website.. http://www.pesticideinfo.org/Detail_ChemReg.jsp?Rec_Id=PC35766.

