# Supplementary material for: Lethal and Pre-Lethal Effects of a Fungal Biopesticide Contribute to Substantial and Rapid Control of Malaria Vectors
Source: PLoS One. 2011 Aug 29;6(8):e23591. doi: 10.1371/journal.pone.0023591 (PMC3163643; doi:10.1371/journal.pone.0023591)
Supplement: Supporting Information S1 — Further details of the material and methods, analyses and results for the experiments described in the main text. (DOC) [file pone.0023591.s001.doc]

**Supporting Information.**

**Fungal preparation and formulation**

Fungal maintenance

## Isolates were maintained in long-term storage at -80^o^C on microporous beads (Pro-Lab Diagnostics, Austin, Texas, USA). Prior to use, the fungus was recovered by placing one or two beads onto Potato dextrose agar (Oxoid, UK) or Sabouraud dextrose agar (Oxoid, UK) in 9 cm diameter Petri dishes or slopes in 25 ml Universal bottles and incubated at 25^o^C for 10 days.

## Mass production

Liquid Culture

Conidia were harvested from slopes or plates to make a spore suspension of approximately 1 x 10^6^ conidia ml^–1^ in sterile 0.05% w/v Tween 80 (Sigma) in distilled water. One ml of this suspension was then used to inoculate 75 ml sterile liquid medium culture medium (4% d-Glucose, 2% yeast extract [Oxoid, UK] in tap water), in 250 ml Erlenmyer flasks. Flasks were incubated on a rotary shaker (160 rpm) at 24^o^C for 3 days.

Solid substrate

Barley flakes (Bobs Red Mill, Milwaukie, Oregon, USA) were weighed into mushroom spawn bags (Unicorn, Garland, Texas, USA), 1 kg per bag and 600 ml tap water was added and the contents mixed by hand to ensure even absorption of the water. The spawn bags were then placed inside autoclave bags for protection and autoclaved for 30 min at 121^o^C. Once cool the bags were inoculated under aseptic conditions with 75 ml of the 4 day old liquid medium plus 75 ml of sterile water to achieve a final moisture content of approximately 48%. The inoculated bags were carefully massaged to ensure even distribution of the inoculum. The bags were then sealed and incubated on shelves for 10 days at 24^o^C. Following incubation, the bags were opened in a reverse flow cabinet (Labconco, USA) and the contents transferred to brown paper bags for drying. The paper bags were placed in a dehumidified room for 4 days (24-30^o^C), until the sporulated substrate reached <20% moisture content. The conidia were then harvested from the barley flakes using a Mycoharvester (Acis Manufacturing, Devon, UK). The harvested conidia were placed in glass dishes and further dried in a desiccator over dry silica gel at 24^o^C. Once the conidia powder reached 5% moisture content, a small sample was taken for quality analysis and the remaining powder was sealed in foil laminated sachets with a small sachet of silica gel and stored at 5^o^C until use.

Germination test

To test the viability of conidia in the formulation prior to spaying, 0.5 ml of the ‘stock’ [or highest concentration ~1 x 10^10^ sp/ml] was taken and diluted in pure Isopar M to a concentration of approximately 1 x 10^7^ sp ml^-1^. One drop of this suspension was transferred onto SDA in 6 cm diameter Petri plates using a microspatula and spread evenly over the surface of the agar. Three plates were prepared for each isolate formulation and incubated at 25^o^C for 20 hr. After incubation, the conidia on the agar surface were examined under a compound microscope (at 500x magnification). Conidia were counted as germinated if a germ was visibly protruding from the conidium and all conidia in each field of view were assessed. A total of at least 300 conidia were counted per plate and viability was calculated as a percentage of the total.

**Mosquito rearing**

*Anopheles stephensi* were reared under standard insectary condition at 27^o^C, 80% humidity and 12 L: 12 D photo-period. Eggs were placed in plastic trays (25 cm x 25 cm x 7 cm) filled with 1.5 l of distilled water. To reduce variation in adult size at emergence, larvae were reared at a fixed density of 400 per tray. Larvae were fed Liquifry for five days and then on Tetrafin fish flakes. From approximately two weeks after egg hatch pupae were collected daily and placed in emergence cages. The adults that emerged were fed *ad libitum* on a 10% glucose solution. All experiments used three to five day old adult female mosquitoes.

**Substrates, fungal formulation, application and mosquito exposure.**

Clay (white earthenware; Clay-King Inc.) tiles were made by mixing the powdered product with water until it reached pouring consistency. The mix was then poured into 150 mm diameter Petri dishes and allowed to set.

Spores of *Beauveria bassiana* isolate IMI39150 used in a number of previous studies (e.g. 1, 2) were suspended in a mix of mineral oils (80% Isopar M: 20% Ondina 22) and the concentration adjusted to give 1x10^9^ spores/ml^-1^. Spores were applied using a pump sprayer clamped horizontally over the test tile 10cm above it. The pump sprayer comprised a pump spray cap (Calmar Inc.) screwed onto a 25ml^-1^ glass universal vial. Each tile received five pumps from the sprayer which delivered 0.7ml ml^-1^ of formulation with each pump which gave an application rate of 3.3x10^11^ spores/m^2^. This standard dose was serially diluted to give application rates of 1.65x10^11^ (50%), 3.3x10^10^ (10%), 1.65x10^10^ (5%) and 3.3x10^9^ (1%) spores/m^2^ (percent of the standard dose) in the assay examining the effects of dose on survival time (see table 1 for full survival results for this assay). Following application tiles were left to dry at room temperature for 24 hours.

A standard WHO cone assay [3] was used for exposing the mosquitoes to the treated tiles. The plastic cone was secured over each tile and between 30 and 60 unfed female *An. stephensi* were introduced. The mosquitoes were left for 30 minutes (see main text) or 10, 5 or 1 minute or 30 seconds in the assay examining the impact of exposure time on survival (see table 2 for full survival results for this assay). Each treatment for all assays was replicated four times giving a minimum of 120 mosquitoes per assay. Following exposure mosquitoes were removed to holding cages where they were either blood fed (see below) or maintained on 10% glucose water for the duration. Mortality was monitored daily and dead insects removed from the cages.

Survival was analyzed with Kaplan-Meier survival analysis in SPSS for Mac v. 18 with differences in median survival times assessed using a Log rank test.

Table S1. Survival estimates for *Anopheles stephensi* exposed to *Beauveria bassiana* at the standard dose (see main text) and serial reductions of the standard dose. All exposures were for 30 minutes. Estimates of time to 100% mortality are the mean of the four replicates in each treatment group. N/A indicates the survival estimate (50% or 100% mortality) was not achieved across the study period.

| **Treatment Group** | **Median Lethal Time days (95% C.I.)*** | **Log Rank Statistic (Significance compared to control)** | **Time to 100% mortality days (± 1 SEM)*** |
| --- | --- | --- | --- |
| **Control** | N/A | --------- | N/A |
| **Standard dose** | 4.0  (3.90-4.10) | 271.7  (*P* < 0.001) | 5.0  (± 0.00) |
| **50%** | 4.0  (3.84-4.16) | 250.7  (*P* < 0.001) | 6.0  (± 0.71) |
| **10%** | 5.0  (4.90-5.10) | 275.5  (*P* < 0.001) | 6.5  (± 0.29) |
| **5%** | 5.0  (4.87-5.14) | 284.8  (*P* < 0.001) | 7.0  (± 0.71) |
| **1%** | 5.0  (4.82-5.18) | 279.3  (*P* < 0.001) | 9.0  (± 0.58) |

Table S2. Survival estimates for *Anopheles stephensi* exposed to clay substrates treated with the standard dose of *Beauveria bassiana*. Exposure times were the standard 30 minutes or for shorter periods as described in the table. Control mosquitoes were exposed for 30 minutes. Estimates of time to 100% mortality are the mean of the four replicates in each treatment group.N/A as described in Table 1.

| **Treatment Group** | **Median Lethal Time days (95% C.I.)*** | **Log Rank Statistic (Significance compared to control)** | **Time to 100% mortality days (± 1 SEM)*** |
| --- | --- | --- | --- |
| **Control** | N/A | --------- | N/A |
| **30 minutes** | 4.0  (3.95-4.05) | 603.7  (*P* < 0.001) | 4.8  (± 0.25) |
| **10 minutes** | 4.0  (3.92-4.08) | 615.9  (*P* < 0.001) | 5.0  (± 0.00) |
| **5 minutes** | 4.0  (3.89-4.11) | 603.4  (*P* < 0.001) | 5.5  (± 0.29) |
| **1 minute** | 4.0  (3.86-4.14) | 540.6  (*P* < 0.001) | 6.0  (± 0.00) |
| **30 seconds** | 4.0  (3.87-4.13) | 583.3  (*P* < 0.001) | 6.3  (± 0.25) |

**Feeding propensity.**

We assessed feeding propensity in two ways. Mosquitoes maintained on glucose water were offered a feeding stimulus comprised of a 250 ml flask filled with hot tap water and covered with the investigators worn sock. This stimulus provides both a heat and odor cue and is a routine technique for sorting female from male *An. stephensi* for experimental purposes in our laboratory. The number observed actively probing through the mesh wall of the cage when the stimulus was placed at the side or the number not moving to the stimulus (whichever was most efficient to count) was recorded and the proportion feeding calculated from the total number in the cage at the time the stimulus was offered. For blood feeds a mouse (C57BL/6J) was anaesthetized and placed on top of a cage and the mosquitoes allowed to feed for 20 minutes. Once the mouse was removed the number of mosquitoes that took a blood meal was recorded and related, as above, to the total number in the cage at the time of the feed. In the first assay looking at blood feeding (“Survival of blood fed and sugar fed mosquitoes and propensity to feed” – see main text results) we removed non-fed and partially fed mosquitoes after the blood fed and hence day 0 proportion feeding is 1 (see Fig 2A). In the “Delayed fungal exposures” results section we did not remove non-fed mosquitoes at their first feed on day 0 and hence the starting proportion feeding varies between 83 and 92% (see Fig 3B). Feeding propensity was analyzed with repeated measures ANOVA (SPSS v.18) with simple contrasts performed to examine difference between treatments for each day.

**Respirometry to determine resting metabolic rate.**

Metabolic rates were measured by using groups of three mosquitoes at rest within flow-through respirometry chambers. Dry CO_2_ free air was passed through the 20 ml chambers at 0.25 litres/min and then dried and passed through a Li-Cor 6252 carbon dioxide analyser. Within each run, seven experimental chambers containing mosquitoes were sampled in sequential fashion by using a computer controlled valve system. Three chambers containing control mosquitoes and four for fungal exposed mosquitoes were used in the first run on each day and the order was reversed for the second run giving 7 replicate estimates per day of the experiment. A base line run was performed on day 0 using the mosquitoes to be allocated to treatment groups and then two runs were made everyday for the duration of the experiment. All chambers were housed in a reach-in incubator set to 25 (± 0.2)^o^C. Analog signals from the flow meter and carbon dioxide analyzer were converted to digital and recorded on a computer (Sable Systems, Salt Lake City). After each run, mosquitoes were removed, killed and their wing lengths measured as an estimate of body size. Analysis was performed on the CO_2_ml^-1^hr^-1^ divided by the total wing length of the three mosquitoes in the chamber. GLM (SPSS v.18) was used to examine differences between treatments with the full model containing block (run 1 or 2), chamber, winglength, day of the experiment and treatment (control and *Beauveria* exposed). Statistical analysis involved fully cross-factored GLMs because no mosquitoes were reused at subsequent time points. There were no significant effects of block (F_1,33_ = 1.40, *P* = 0.25), chamber (F_1,33_ = 0.81, *P* = 0.37), winglength (F_1,33_ = 0.17, *P* = 0.69) or day (F_2,33_ = 0.49, *P* = 0.62).

**Flight assays.**

To assess flight performance female mosquitoes were briefly immobilized with CO_2_ and then placed on a dish under a dry ice curtain. A small amount of dental wax was melted on the blade of a surgical scalpel and the head of an insect pin (Bioquip products, Inc No. 2 pin) touched to the wax. Immediately following, a mosquito gently held in forceps was manipulated so that the dorsal thorax came into contact with the head of the pin, held briefly to allow the wax to set, and then the sharp end of the pin was stuck vertically in a block of polystyrene. Each mosquito was carefully examined to ensure that no wax had spread over the insect, that no damage to the insect had been caused and that the insects’ orientation was appropriate (i.e. not cantered to one side, forward or back). In any case where the mounting of the insect on the pin did not satisfy these requirements the mosquito was discarded and a new one set up. We aimed to set-up ten mosquitoes from each group (control and *Beauveria* exposed). Two measures were made: 1) the duration of voluntary flight. In this case the time until first flight was initiated by the mosquito was recorded as well as the duration of this flight. 2) The second measure looked at the duration of sustained flight. In this case and following the end of the voluntary flight the mosquito was stimulated to fly again by gently stroking its legs with a fine paint brush. Each time the mosquito stopped flying it was stimulated again until after five consecutive stimulations the insect could not fly again. Mosquitoes were exposed to fungal treated clay tiles for the same duration and at the same dose as described above. Control mosquitoes were exposed to untreated tiles. Flight performance was analyzed using Mann-Whitney U Test (SPSS v.18) with Treatment as the grouping variable and time to flight/flight time for each day after exposure as the test variable. Only animals that flew at all were included in the statistical analyses. Full statistical outputs can be found in tables 3, 4 and 5.

Table S3. Mann-Whitney U statistics examining the effect of Beavueria bassiana exposure on time to initiate voluntary flight by *Anopheles stephensi*.

|  | **Time to initiate voluntary flight** | | | |
| --- | --- | --- | --- | --- |
|  | Mann-Whitney U Test statistic | Sample size | Z value | Significance |
| **Day 1** | 13.0 | 14 | -1.57 | *P* = 0.12 |
| **Day 2** | 11.0 | 18 | -2.66 | *P* = 0.008 |
| **Day 3** | 11.0 | 18 | -2.75 | *P* = 0.009 |
| **Day 4** | 14.0 | 18 | -2.34 | *P* = 0.019 |

Table S4. Mann-Whitney U statistics examining the effect of Beavueria bassiana exposure on the duration of voluntary flight by *Anopheles stephensi*.

|  | **Duration of voluntary flight** | | | |
| --- | --- | --- | --- | --- |
|  | Mann-Whitney U Test statistic | Sample size | Z value | Significance |
| **Day 1** | 16.0 | 14 | -1.09 | *P* = 0.28 |
| **Day 2** | 7.0 | 18 | -2.94 | *P* = 0.003 |
| **Day 3** | 9.0 | 18 | -2.76 | *P* = 0.004 |
| **Day 4** | 10.0 | 13 | -1.47 | *P* = 0.17 |

Table S5. Mann-Whitney U statistics examining the effect of Beauveria bassiana exposure on the duration of sustained flight by *Anopheles stephensi*.

|  | **Duration of sustained (stimulated) flight** | | | |
| --- | --- | --- | --- | --- |
|  | Mann-Whitney U Test statistic | Sample size | Z value | Significance |
| **Day 1** | 18.0 | 14 | -0.85 | *P* = 0.40 |
| **Day 2** | 35.5 | 17 | -0.051 | *P* = 0.96 |
| **Day 3** | 10.0 | 15 | -2.07 | *P* = 0.039 |
| **Day 4** | 3.0 | 12 | -2.44 | *P* = 0.015 |

**Assays against insecticide resistant mosquitoes.**

These assays were carried out at the Vector Control Reference Unit, National Institute for Communicable Diseases, National Health Laboratory Service, Johannesburg South Africa. The unit has a unique variety of *Anopheles* colonies collected from various African countries and with documented susceptibility profiles to chemical insecticides. A list of the colonies, the species, their origin and time as an insectary colony can be found in table 6.

Two separate assays were performed dictated by the availability of suitable numbers for the colonies at the time. For each exposure assay a fully susceptible *An. gambiae* s.s. (SUA) colony was included as a positive control. The standard dose (above) was used and formulation, spray application and mosquito exposure were all also as described above. Mosquitoes for this assay were housed in 0.375 liter cardboard cups covered with mesh lids and provided with an *ad libitum* supply of 10% sucrose via a soaked cotton pad placed on the mesh. Mortality was assessed daily and cadavers removed. Both assays were run for 14 days. Median lethal times, time to 100% mortality and the significance compared to the control for each colony are shown in table 7. For colonies COGS-BASE and COGS-EXPO mosquito numbers were limited and hence each treatment group was replicated three times with approximately 20 mosquitoes per replicate.

Following the fungal assays mosquitoes were drawn from each colony and exposed to four chemical insecticides representing the range of compounds currently in use for mosquito control. These chemical compounds are organochlorides, organophosphates, pyrethroids and carbamates for which we used DDT, malathion, deltamethrin and bendiocarb commercial insecticides respectively. Chemical assays were conducted using WHO plastic cylinders and carried out according to their guidelines [3]. Test papers were treated with the discriminatory dose of each insecticide as prescribed by WHO [4]. In brief 25-30 mosquitoes were aspirated into a WHO test cylinder which had been lined with test paper treated with the discriminatory dose approved for that chemical. After one hour mosquitoes were blown into the opposite end of the tube containing untreated papers and supplied with 10% sucrose via a cotton pad. Twenty four hours late mortality was scored. Dose rates, percent mortality and the susceptibility rating to the chemical are shown in table 8.

Table S6. Descriptions and origin for mosquito colonies used in the fungal assays against insecticide resistant mosquitoes.

| **Mosquito species** | **Colony Name** | **Field origin** | **Started in Culture** |
| --- | --- | --- | --- |
| *An. gambiae* s.s. | SUA | Suakoko, Liberia | Unknown |
| *An. arabiensis* | SENN-base | Sennar, Sudan | 1980’s |
| *An. arabiensis* | SENN-DDT | Sennar, Sudan | 2004 |
| *An arabiensis* | MBN-Base | Mamfene, South Africa | 2001 |
| *An arabiensis* | MBN-DDT | Mamfene, South Africa | 2003 |
| *An. funestus* | FUMOZ-Base | Southern Mozambique | 2000 |
| *A. funestus* | FUMOZ-R | Southern Mozambique | 2000 |
| *An. gambiae* | SOG | Obuasi, Ghana | 2004 |
| *An. gambiae* | BENROG | Obuasi, Ghana | 2005 |
| *An. gambiae* | GAH | Ahafo, Ghana | 2004 |
| *An. gambiae* | COGS-Expo | Pointe Noire, Republic of the Congo | 2009 |
| *An. gambiae* | COGS-Base | Pointe Noire, Republic of the Congo | 2009 |
| *An. gambiae* | TONGS | Tongon, Cote d’Ivoire | 2010 |

Table S7. Mortality twenty four hours after a one hour exposure to one of four insecticides each representing a different chemical compound Dose rates the discriminatory dose prescribed by WHO for testing insecticide resistance. Percent mortality (± 1 S.D.) is shown and letters following indicate the degree of resistance shown to the chemical: S = fully susceptible (97-100% mortality), R/S = resistance suspected (80-96% mortality) and R = resistant (< 80% mortality).

| **Mosquito species** | **Colony Name** | **Insecticide susceptibility (Percent mortality after 24 hours ± 1 S.D.) and insecticide susceptibility rating.** | | | |
| --- | --- | --- | --- | --- | --- |
|  |  | **DDT 4%** | **Deltamethrin 0.05%** | **Malathion 5%** | **Bendiocarb 0.1%** |
| *An. gambiae* | SUA | 100 (±0.0)  S | 100 (±0.0)  S | 100 (±0.0)  S | 100 (±0.0)  S |
| *An. arabiensis* | SENN BASE | 100.0 (±0.0)  S | 95.4 (± 1.83)  R/S | 97.9 (±3.61)  S | 100 (±0.0)  S |
| *An arabiensis* | MBN BASE | 80.95 (±23.4)  R/S | 92.82 (±3.11)  R/S | 20.7 (±6.95)  R | 100 (±0.0)  S |
| *An arabiensis* | MBN DDT | 2.7 (±2.32)  R | 28.1 (±14.7)  R | 17.9 (±3.78)  R | 100 (±0.00)  S |
| *An. funestus* | FUMOZ BASE | 100 (±0.00)  S | 3.0 (±5.25)  R | 100 (±0.00)  S | 60.1 (±7.87)  R |
| *A. funestus* | FUMOZ R | 1. (±0.00)   S | 1. (±0.00)   R | 98.4 (±2.75)  S | 51.1 (±11.2)  R |
| *An. arabiensis* | SENN DDT | 1. (±0.00)   R | 13.0 (±5.05)  R | 60.0 (±10.53)  R | 100 (±0.00)  S |
| *An. gambiae* | SOG | 64.4 (±14.57)  R | 82.1 (±4.35)  R/S | 100 (±0.00)  S | 100 (±0.00)  S |
| *An. gambiae* | BENROG | 100 (±0.00)  S | 100 (±0.00)  S | 100 (±0.0)  S | 61.7 (±11.8)  R |
| *An. gambiae* | GAH | 71.8 (±6.54)  R | 83.8 (±9.47)  R/S | 95.3 (±0.33)  R/S | 98.4 (±2.75)  S |
| *An. gambiae* | COGS EXPO | 1.5 (±2.51)  R | 82.5 (±15.4)  R/S | 100 (±0.00)  S | 100 (±0.00)  S |
| *An. gambiae* | COGS BASE | 5.8 (±5.12)  R | 94.6 (±5.8)  R/S | 100 (±0.00)  S | 100 (±0.00)  S |
| *An. gambiae* | TONGS | 1.5 (±2.51)  R | 8.8 (±4.35)  R | 76.6 (±7.05)  R | 46.1 (±19.0)  R |

Table S8. Survival estimates for different *Anopheles* colonies exposed to *Beauveria bassiana* at the standard dose (see main text). All exposures were for 30 minutes. Estimates of time to 100% mortality are the mean of the four replicates in each treatment group. N/A indicates the survival estimate (50% or 100% mortality) was not achieved across the study period. Refer to Table 6 for more information on each mosquito colony.

| **Mosquito colony** | **Treatment** | **Median Lethal time (± 95% C.I.) days** | **Mean time to 100% mortality (± 1 SEM) days** | **Log Rank Statistic** | **Significance** |
| --- | --- | --- | --- | --- | --- |
| **Assay 1** | | | | | |
| **SUA** | **Control** | N/A | --------- | 228.7 | *P* < 0.001 |
|  | ***Beauveria*** | 4.0  (3.82-4.18) | 6.25  (±0.19) |  |  |
| **TONGS** | **Control** | N/A | -------- | 218.0 | *P* < 0.001 |
|  | ***Beauveria*** | 4.0  (3.93-4.07) | 5.0  (± 0.0) |  |  |
| **MBN-BASE** | **Control** | 13.0  (11.40-14.60) | --------- | 186.1 | *P* < 0.001 |
|  | ***Beauveria*** | 3.0  (2.79-3.21) | 4.0  (± 0.0) |  |  |
| **MBN-DDT** | **Control** | N/A | --------- | 179.8 | *P* < 0.001 |
|  | ***Beauveria*** | 3.0  (2.87-3.13) | 4.5  (± 0.29) |  |  |
| **FUMOZ-BASE** | **Control** | N/A | ---------- | 225.2 | *P* < 0.001 |
|  | ***Beauveria*** | 4.0  (3.95-4.05 | 4.5  (± 0.29) |  |  |
| **FUMOZ-R** | **Control** | N/A | --------- | 192.2 | *P* < 0.001 |
|  | ***Beauveria*** | 4.0  (3.90-4.10) | 5.0  (± 0.0) |  |  |
| **Assay 2** | | | | | |
| **SUA** | **Control** | N/A | --------- |  |  |
|  | ***Beauveria*** | 4.0  (3.82-4.18) | 6.0  (± 0.41) | 199.2 | *P* < 0.001 |
| **SENN-BASE** | **Control** | N/A | --------- | 238.2 | *P* < 0.001 |
|  | ***Beauveria*** | 4.0  (3.92-4.08) | 5.5  (± 0.5) |  |  |
| **SENN-DDT** | **Control** | N/A | --------- | 250.7 | *P* < 0.001 |
|  | ***Beauveria*** | 4.0  (3.82-4.18) | 6.25  (± 0.25) |  |  |
| **SOG** | **Control** | N/A | --------- | 203.5 | *P* < 0.001 |
|  | ***Beauveria*** | 4.0  (3.96-4.04) | 4.5  (± 0.29) |  |  |
| **BENROG** | **Control** | N/A | --------- | 207.9 | *P* < 0.001 |
|  | ***Beauveria*** | 4.0  (3.95-4.05) | 4.75  (± 0.48) |  |  |
| **GAH** | **Control** | 8.0  (6.43-9.57) | --------- | 102.3 | *P* < 0.001 |
|  | ***Beauveria*** | 4.0  (3.91-4.09) | 5.5  (± 0.29) |  |  |
| **COGS-BASE** | **Control** | 12.0  (8.90-15.01) | --------- | 9.82 | *P* = 0.002 |
|  | ***Beauveria*** | 4.0  (1.89-6.11) | 5.3  (± 0.88) |  |  |
| **COGS-EXPO** | **Control** | 8.0  (2.77-13.23) | --------- | 10.9 | *P* = 0.001 |
|  | ***Beauveria*** | 4.0  (2.83-5.17) | 6.0  (± 0.58) |  |  |

References.

1. Blanford S, et al. (2005) Fungal pathogen reduces potential for malaria transimission. *Science* 308: 1638-1641.
2. Bell AS, Blanford S, Jenkins N, Thomas MB, Read AF (2009) Real-time quantitative PCR analysis of candidate fungal biopesticides against malaria: Technique validation and first applications. J Invert Pathol 100: 160-168.
3. World Health Organisation website Available at: <http://whqlibdoc.who.int/hq/2006/WHO_CDS_NTD_WHOPES_GCDPP_2006.3_eng.pdf>. Accessed 2011 Aug 1.
4. World Health Organisation website. Available at: <http://whqlibdoc.who.int/hq/2006/WHO_CDS_NTD_WHOPES_GCDPP_2006.3_eng.pdf> Accessed 2011 Aug 1.
